# Vitamin D Supplementation Reduces Induction of Epithelial-Mesenchymal Transition in Allergen Sensitized and Challenged Mice

**DOI:** 10.1371/journal.pone.0149180

**Published:** 2016-02-12

**Authors:** Kimberly D. Fischer, Sannette C. Hall, Devendra K. Agrawal

**Affiliations:** 1 Department of Medical Microbiology and Immunology, Creighton University School of Medicine, Omaha, NE, United States of America; 2 Department of Biomedical Science, Creighton University School of Medicine, Omaha, NE, United States of America; 3 Department of Clinical and Translational Science, Creighton University School of Medicine, Omaha, NE, United States of America; National Jewish Health, UNITED STATES

## Abstract

Asthma is a chronic disease of the lung associated with airway hyperresponsiveness (AHR), airway obstruction and airway remodeling. Airway remodeling involves differentiation of airway epithelial cells into myofibroblasts via epithelial-mesenchymal transition (EMT) to intensify the degree of subepithelial fibrosis. EMT involves loss in E-cadherin with an increase in mesenchymal markers, including vimentin and N-cadherin. There is growing evidence that vitamin D has immunomodulatory and anti-inflammatory properties. However, the underlying molecular mechanisms of these effects are still unclear. In this study, we examined the contribution of vitamin D on the AHR, airway inflammation and expression of EMT markers in the airways of mice sensitized and challenged with a combination of clinically relevant allergens, house dust mite, ragweed, and *Alternaria* (HRA). Female Balb/c mice were fed with vitamin D-sufficient (2000 IU/kg) or vitamin D-supplemented (10,000 IU/kg) diet followed by sensitization with HRA. The density of inflammatory cells in the bronchoalveolar lavage fluid (BALF), lung histology, and expression of EMT markers by immunofluorescence were examined. Vitamin D-supplementation decreased AHR, airway inflammation in the BALF and the features of airway remodeling compared to vitamin D-sufficiency in HRA-sensitized and -challenged mice. This was accompanied with increased expression of E-cadherin and decreased vimentin and N-cadherin expression in the airways. These results indicate that vitamin D may be a beneficial adjunct in the treatment regime in allergic asthma.

## Introduction

Chronic inflammation in the airways causes structural and functional changes in the lungs that may result in asthma. Physiological changes in asthma include narrowing of the airways following exposure to allergens or bronchoconstrictors, leading to airway hyperresponsiveness (AHR). Structural changes feature the characteristics of airway remodeling including epithelial cell shedding, goblet cell hyperplasia/metaplasia, subepithelial fibrosis, smooth muscle cell hyperplasia, edema, and angiogenesis [1]. Current therapies, such as corticosteroids, leukotriene antagonists, and long-acting β2 agonists, may be effective in dampening inflammation, but are ineffective in preventing or reversing airway remodeling [2], [3]. Additionally, therapies such as corticosteroids may induce apoptosis in epithelial cells, thereby contributing to epithelial shedding [4]. Thus, additional consideration must be taken to understand the cause of airway remodeling in order to acquire therapies that target molecules involved in structural alterations, including subepithelial fibrosis and epithelial thickening. The process of airway remodeling involves the release of inflammatory mediators from immune cells in the airway, including transforming growth factor (TGF)-β, tumor necrosis factor (TNF)-α, interleukin (IL)-4, and IL-13 with the disruption of the epithelium and subepithelial fibrosis [5].

Initiation of subepithelial fibrosis in the airway epithelial cell can occur through activation of pathways resulting into epithelial mesenchymal transition (EMT). Activation of EMT signaling causes the cells to differentiate into myofibroblasts, enabling invasion and migration within the epithelial layer. Increased myofibroblasts in the sub-mucosa secrete collagen and extracellular matrix, thereby contributing to the subepithelial fibrosis in airway remodeling [6].

A number of studies have examined the relationship between vitamin D and asthma with a growing body of evidence suggesting that vitamin D deficiency is linked with the pathogenesis of asthma [7]. Treatment of asthma though oral supplementation of vitamin D has been not well studied and has given mixed results in terms of patient outcomes, as recently reviewed by Yawn and colleagues [8]. Further understanding of how vitamin D functions are necessary to provide therapeutic guidelines for the use of vitamin D to control the pathogenesis of asthma.

Mouse models of allergic airway inflammation and AHR are generally used to determine the cellular and molecular mechanisms. Most often, ovalbumin (OVA) is used as an allergen. However, sometimes it is difficult to extrapolate the findings to human unless the studies are performed with clinically-relevant allergens [9]. Such allergens have been used sparingly in animal models of allergic airway inflammation. House dust mite [10], ragweed [11], and *Alternaria alternata* extracts [12] have been used individually in mouse models, but never in combination with one another. This study utilizes the combination of these allergens in a mouse model of asthma while evaluating the induction of EMT protein markers in the lung.

## Materials and Methods

### Animals and Diets

Female and male BALB/c mice were purchased from Harlan Laboratories (Indianapolis, IN). Mice were maintained in a specific pathogen-free environment at the Animal Resource Facility of Creighton University and monitored daily. The Institutional Animal Care and Use Committee of Creighton University approved the research protocol of this study. Food and water were provided *ad libitum*. Vitamin D supplemented feed and normal mouse feed containing sufficient vitamin D levels were given to breeding male and female mice. Female offspring were weaned on the same diets consisting of normal vitamin D levels of 2,000 IU/kg and vitamin D-supplemented diet levels of 10,000 IU/kg (Harlan Laboratories, Madison, WI). No animals during the experimental procedure became ill or died prior to the experimental endpoint but were monitored before and during the experiment. Mice were monitored for distress, through the lack of movement, difficulty in breathing, and rolling on the back. If the event of any distress, the animal would be taken out of the cage and examined. If the distress were found to be due to a respiratory problem, albuterol would be administered by aerosol. In this study, none of the animals were found to be under distress at any time.

### Induction of Allergic Airway Disease

**Fig 1:**
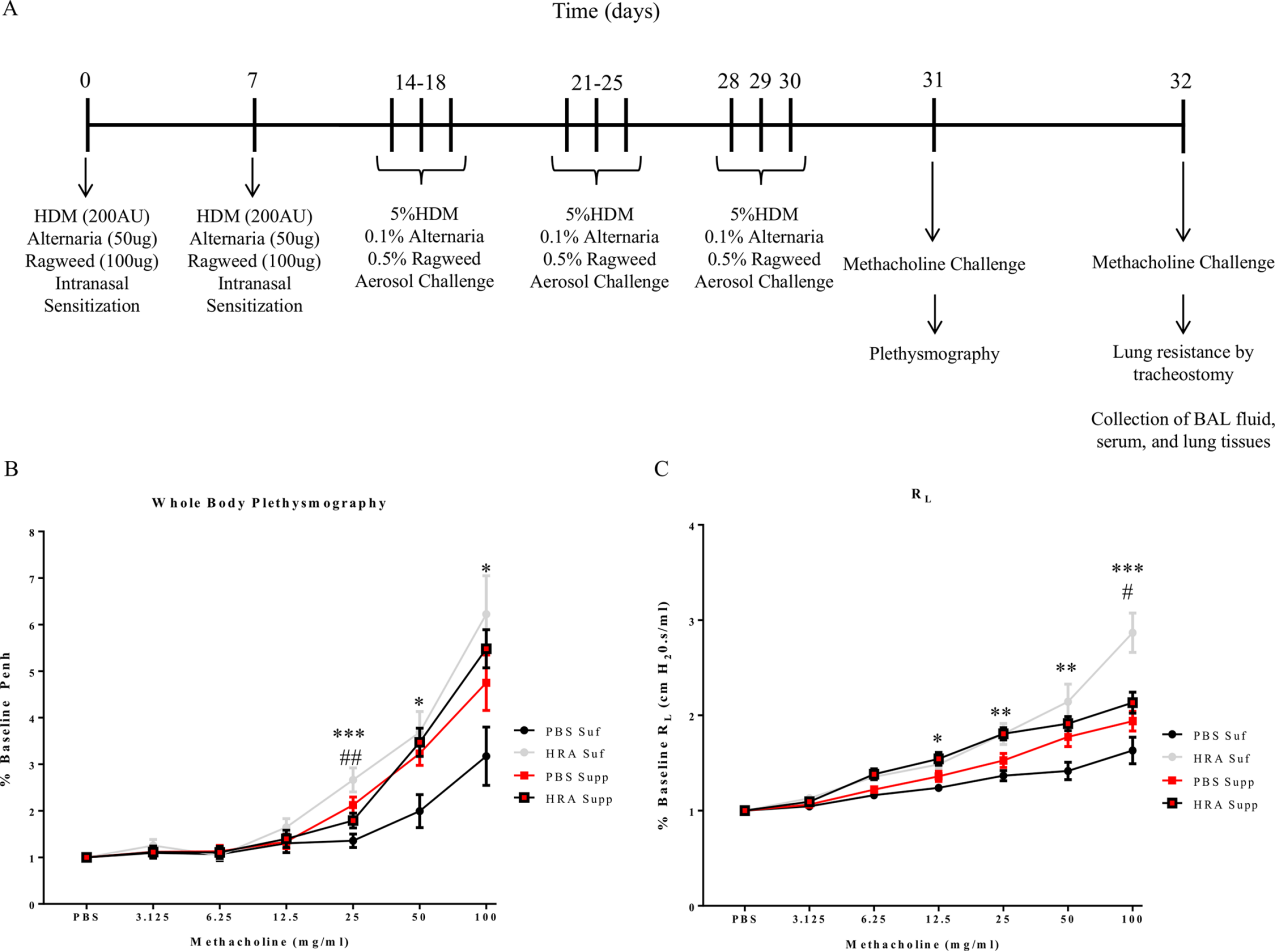
Protocol for HRA-sensitization and challenge in mice and pulmonary function analysis. (A) Mice were divided into vitamin D-sufficient and supplemented groups and then further divided into sensitized and non-sensitized groups. HRA sensitized and challenged model of allergic airway inflammation was established by intranasal installation of 200 allergy units of HDM, 20 μg of Alternaria extract, and 100 μg of ragweed extract. Animals received aerosol challenges of 5% HDM, 0.1% Alternaria, and 0.5% ragweed 3 days a week for 3 weeks from days 14–30. On day 31, airway hyper-responsiveness (AHR) to methacholine was measured by whole body plethysmography (WBP). On day 32, specific lung resistance (R_L_) was measured by tracheostomy; (B) Graph showing the results of enhanced pause (Penh), a measurement of AHR by WBP. The results are expressed as a percentage of baseline Penh value (baseline is defined as 1) and presented as mean ± SEM of 7 mice per group. Vitamin D-sufficient PBS-sensitized and challenged mice vs Vitamin D-sufficient HRA-sensitized and challenged mice (*p <0.05, ***p <0.001); Vitamin D sufficient HRA-sensitized and challenged mice vs. Vitamin D-supplemented HRA-sensitized and challenged mice (## p <0.01); (C) Five randomly selected animals from each group were subjected to a methacholine dose response using invasive tracheostomy and R_L_ was recorded. The results are expressed as a percentage of baseline R_L_ value (baseline is defined as 1) and presented as mean ± SEM of 5 mice per group. Vitamin D-sufficient PBS control mice vs Vitamin D-sufficient HRA-sensitized and -challenged mice (*p <0.05, **p <0.01, ***p < 0.001); Vitamin D- sufficient HRA-sensitized and -challenged mice vs Vitamin D-supplemented HRA-sensitized and -challenged mice (#p <0.05).

Mice 3–4 weeks in age were divided into sensitized and non-sensitized groups. HRA sensitized and challenged model of allergic airway inflammation was established by intranasal installation of 200 allergic units of HDM, 50 μg of *Alternaria alternata* extract, and 100 μg of ragweed allergen extract (Jubilant HollisterStier Allergy, Spokane, WA). Animals received aerosol challenges of mixed antigen containing 5% HDM, 0.1% *Alternaria*, and 0.5% ragweed (HRA) 3 days a week for 3 weeks from days 14–30 (Fig 1A). On day 31, AHR to methacholine was measured by whole body plethysmography (Buxco FinePointe Data Sciences International St. Paul, MN.). On day 32, mice anesthetized using sodium pentobarbital were tracheostomized and placed in a plethysmograph single chamber (Buxco FinePointe Series Resistance and Compliance (RC) Buxco Electronics, Troy, NY). Specific lung specific resistance (R_L_) was measured with increasing doses of nebulized methacholine to measure AHR as previously described [13].

### Bronchoalveolar Lavage Fluid and Cytokine Measurement

Immediately after euthanization of mice using 100 mg/kg dose of sodium pentobarbital i.p., samples of blood and bronchoalveolar lavage fluid (BALF) were collected from the mice. Blood was collected from the left ventricle of mice, allowed to coagulate, serum separated, and stored at -80°C for future analysis. Mouse lungs were then gently lavaged with 1 ml of PBS, centrifuged at 400 × *g* for 10 minutes, and the supernatants stored at −80°C. Total cell counts were performed using the Countess Automated Cell Counter (Invitrogen, Grand Island, NY). Cytospin slides were obtained using a Shandon Cytospin 4 centrifuge machine (Thermo Electron, Waltham, MA). The slides were dried and the cells were fixed and stained using Diff-Quik staining reagent (StatLab Medical Products, Lewisville, TX) according to the manufacturer's instructions. Randomly, a minimum of 300 cells were examined per cytospin slide and absolute cell numbers for each cell type were calculated according to the total leukocyte counts in BALF and the percentage of each individual cell type on the slide. ELISA measured the IgE levels in both serum and BALF according to the manufacturer's instructions using mouse IgE ELISA Detection Ready-Set-Go kit (eBioscience, San Diego, CA).

### Preparation and Staining of Lung Tissue

Lung lobes were fixed in 4% formalin and paraffin embedded. Lung lobes were removed, fixed in 4% formalin and embedded in paraffin in an automatic tissue processer. Lung tissues were sectioned to 4 μm thickness, rehydrated, and stained with hematoxylin and eosin (H&E) following manufacturer’s instructions (Fisher HealthCare PROTOCOL, Waltham, MA). Mucus secretion was identified by periodic acid-Schiff (PAS) reaction (Sigma-Aldrich, St. Louis, MO). Trichrome staining (Thermo Scientific Richard-Allan Scientific, Waltham, MA) was used to identify collagen deposition. Morphometric analysis of goblet cell was performed by counting cells in PAS-stained sections and expressed as the mean number of goblet cells per 100 μm of basal lamina measured by ImageJ image analysis software (National Institutes of Health) [14]. Fibrosis was also quantified by image analysis using ImageJ software. Briefly, the peribronchial fibrosis was traced, converted to grayscale, threshold was adjusted, and the perimeter of the airway was measured. Subepithelial fibrosis is thus expressed as area of fibrosis (μm^2^) per micrometer of basement membrane [15].

### Immunofluorescence

Paraffin embedded lung section slides deparaffinization, rehydration, and underwent antigen retrieval before immunostaining. Non-specific binding was blocked using a solution of 5% normal goat serum, with 0.25% Triton X-100 in PBS. The lung sections were incubated with primary antibodies to E-cadherin [(ab15148) rabbit, Abcam, Cambridge, MA, 1/500 dilution], N-cadherin [(ab18203) rabbit, Abcam, Cambridge, MA, 1/100 dilution], and vimentin [(ab92547) rabbit, Abcam, Cambridge, MA, 1/200 dilution]. The sections were washed and incubated with Alexa Fluor-594 goat anti-rabbit IgG secondary antibody [(DI-1549) Vector Laboratories, Burlingame, CA, 1/500 dilution] and nuclei were counterstained with DAPI. Fluorescent analysis was performed using an Olympus inverted fluorescence microscope (Olympus BX51) and mean fluorescent intensity was measured using ImageJ software (http://rsbweb.nih.gov/ij/). Intensity measurements were performed by analyzing the region of interest specifically to the airway and setting a threshold according to guidelines previously set [16]. The mean fluorescent intensity from the airway was analyzed using the equation corrected total fluorescence (CTF) = integrated density-(area of region of interest x mean fluorescence of background readings) [17]. Negative controls of tissue sections were run without incubation with the primary antibody.

### Measurement of serum 25(OH) D levels

Blood was collected from the left ventricle of vitamin D-deficient and vitamin D-sufficient mice. Serum was separated and the samples were analyzed for the measurement of 25(OH)D levels by ELISA according to the manufacturer's instructions using mouse 25(OH)D kit (MyBioSource, Inc, San Diego, CA).

### Statistical Analysis

Values of all measurements are reported as mean ± SEM. Multiple group comparison was performed using one-way analysis of variance with a Tukey post–hoc test. GraphPad Prism v6.0 was used to analyze data with a *p* value of <0.05 considered significant.

## Results

### Effect of Vitamin D on AHR and Inflammatory Cells in HRA-sensitized and Challenged Mice

Following the protocol shown in Fig 1A, HRA-sensitized and challenged mice demonstrated AHR to methacholine as determined by noninvasive whole-body plethysmography (WBP) (Fig 1B) and invasively (Fig 1C) by measuring specific airway resistance via tracheostomy. Vitamin D-sufficient HRA-sensitized and challenged mice demonstrated significantly elevated AHR to methacholine compared to PBS control mice on a vitamin D-sufficient diet as demonstrated via WBP (Fig 1B) and R_L_ (Fig 1C) for doses 25–100 mg/ml and 12.5–100 mg/ml respectively. HRA-sensitized mice fed with a vitamin D-supplemented diet exhibited significant reduction in AHR compared to vitamin D-sufficient HRA-sensitized mice at 25 mg/ml (Fig 1B) and 100 mg/ml of methacholine (Fig 1C). There was no significant difference in AHR among PBS control vitamin D-sufficient, and PBS control vitamin D-supplemented groups. Aerosolized administration of 100 mg/ml methacholine at day 31 had the following Penh values: 6.223 ± 0.6265 in vitamin D-sufficient HRA-sensitized and challenged mice; 3.172 ± 0.6265 in vitamin D-sufficient PBS-sensitized and challenged mice; 4.75 ± 0.5951 in vitamin D- supplemented HRA-sensitized and challenged mice; 5.481 ± 0.4100 in vitamin D- supplemented PBS-sensitized and challenged mice (n = 7 in each group) (Fig 1B).

Specific airway resistance induced by 100 mg/ml methacholine exhibited mean values of 2.868 ± 0.2086 cm H_2_O.s/ml in vitamin D-sufficient HRA-sensitized and challenged mice; 1.63 ± 0.1404 cm H_2_O.s/ml in vitamin D-sufficient PBS-sensitized and challenged mice; 2.134 ± 0.1105 cm H_2_O.s/ml in vitamin D-supplemented HRA-sensitized and challenged mice; and 1.938 ± 0.1033 cm H_2_O.s/ml in vitamin D-supplemented PBS-sensitized and challenged mice (n = 5 in each group) (Fig 1C).

**Fig 2:**
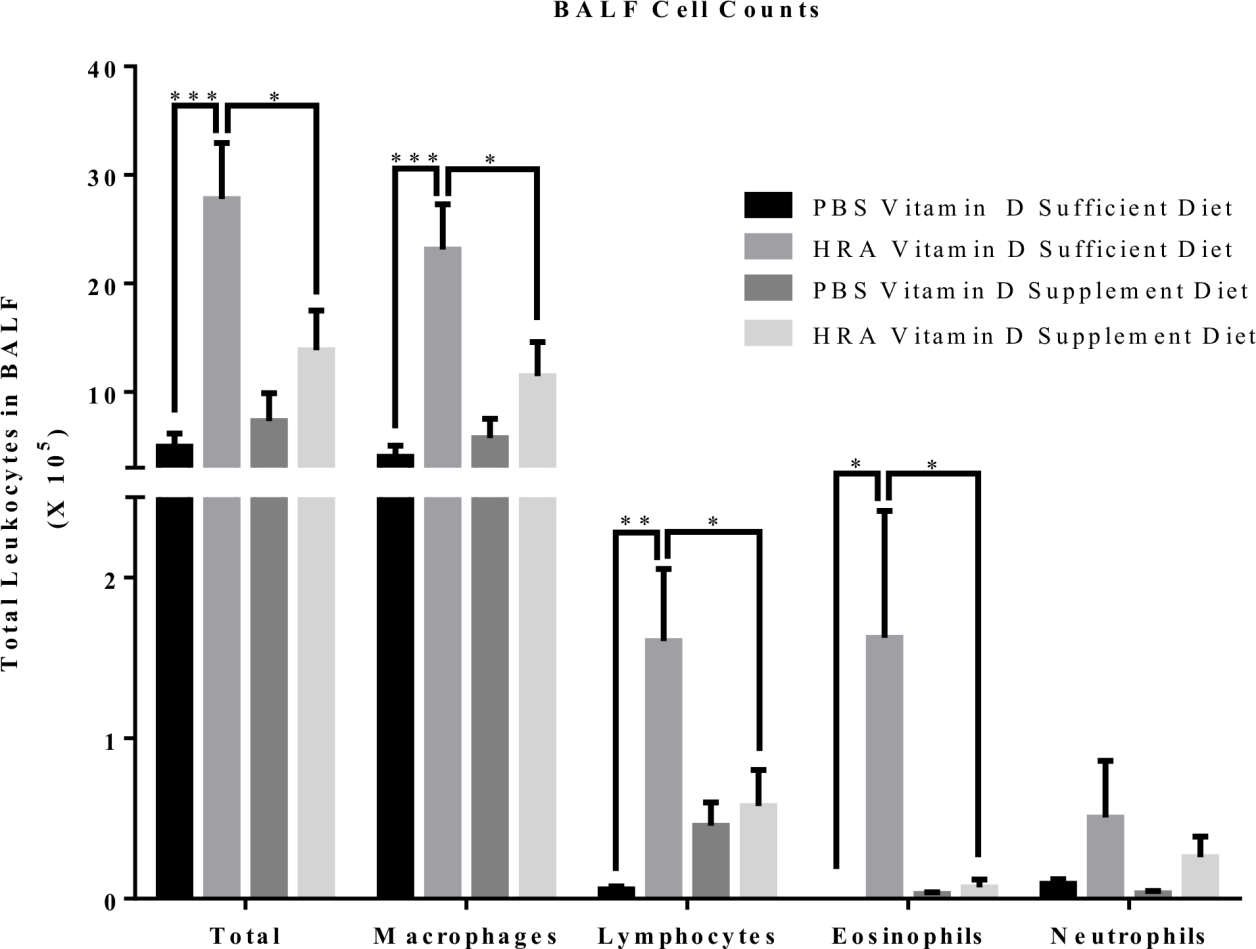
Effect of vitamin D on total and differential leukocytes in BALF of PBS and HRA-sensitized mice. Total cells in the BALF were counted using Countess Automated Cell Counter and differential analysis was performed using standard morphological criteria. A minimum of 300 cells were examined per cytospin slide and absolute cell numbers for each cell type were calculated according to the total leukocyte counts in BALF and the percentage of each individual cell type on the slide. Data are shown as mean ± SEM for seven animals in each group (*p < 0.05; **p < 0.01; ***p < 0.001).

Vitamin D-sufficient HRA-sensitized and challenged mice demonstrated significantly increased total number of cells, macrophages, lymphocytes, and eosinophils compared to vitamin D-sufficient PBS-control mice as well as vitamin D-supplemented PBS and HRA-sensitized and challenged mice (Fig 2). No significant difference was observed in the number of neutrophils between groups.

### Assessment of IgE and Vitamin D Levels in Vitamin D-sufficient and Vitamin D-supplemented Mice

**Fig 3:**
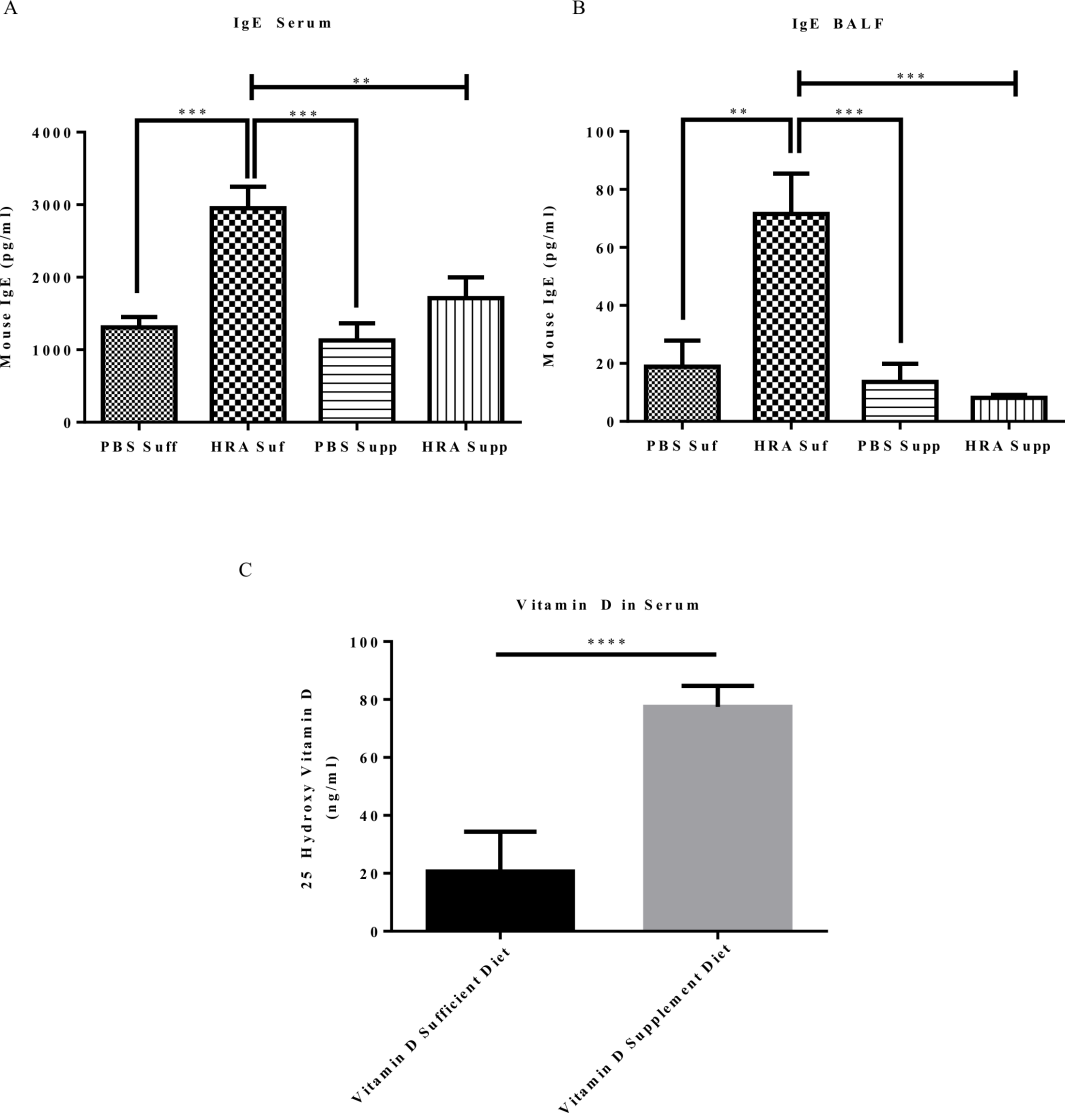
Effect of serum and BALF IgE levels and serum vitamin D levels in vitamin D-sufficient and vitamin D-supplemented mice. (A) Measurement of IgE in mouse serum. The results are presented as mean ± SEM of 7 mice per group, **p < 0.01; ***p < 0.001; (B) Measurement of IgE in mouse BALF. The results are presented as mean ± SEM of 7 mice per group, **p < 0.01; ***p < 0.001; (C) Serum levels of 25(OH)D in vitamin D-sufficient and vitamin D-supplemented mice. The results are presented as ± SEM of 7 mice per group (****p <0.0001).

The levels of IgE in serum and in the BALF of HRA-sensitized and challenged mice were significantly increased in vitamin D-sufficient group compared to vitamin D-sufficient PBS-sensitized and challenged as well as vitamin D-supplement PBS and HRA-sensitized and challenged mice. Therefore, mice on a supplemented vitamin D diet had decreased levels of IgE in both serum (Fig 3A) and in the BALF (Fig 3B). The serum 25(OH)D levels in vitamin D-supplement mice were significantly higher than in the vitamin D-sufficient group mice (Fig 3C).

### Effect of Vitamin D Status on Differences in the Degree of Airway Remodeling After HRA Sensitization and Challenge

**Fig 4:**
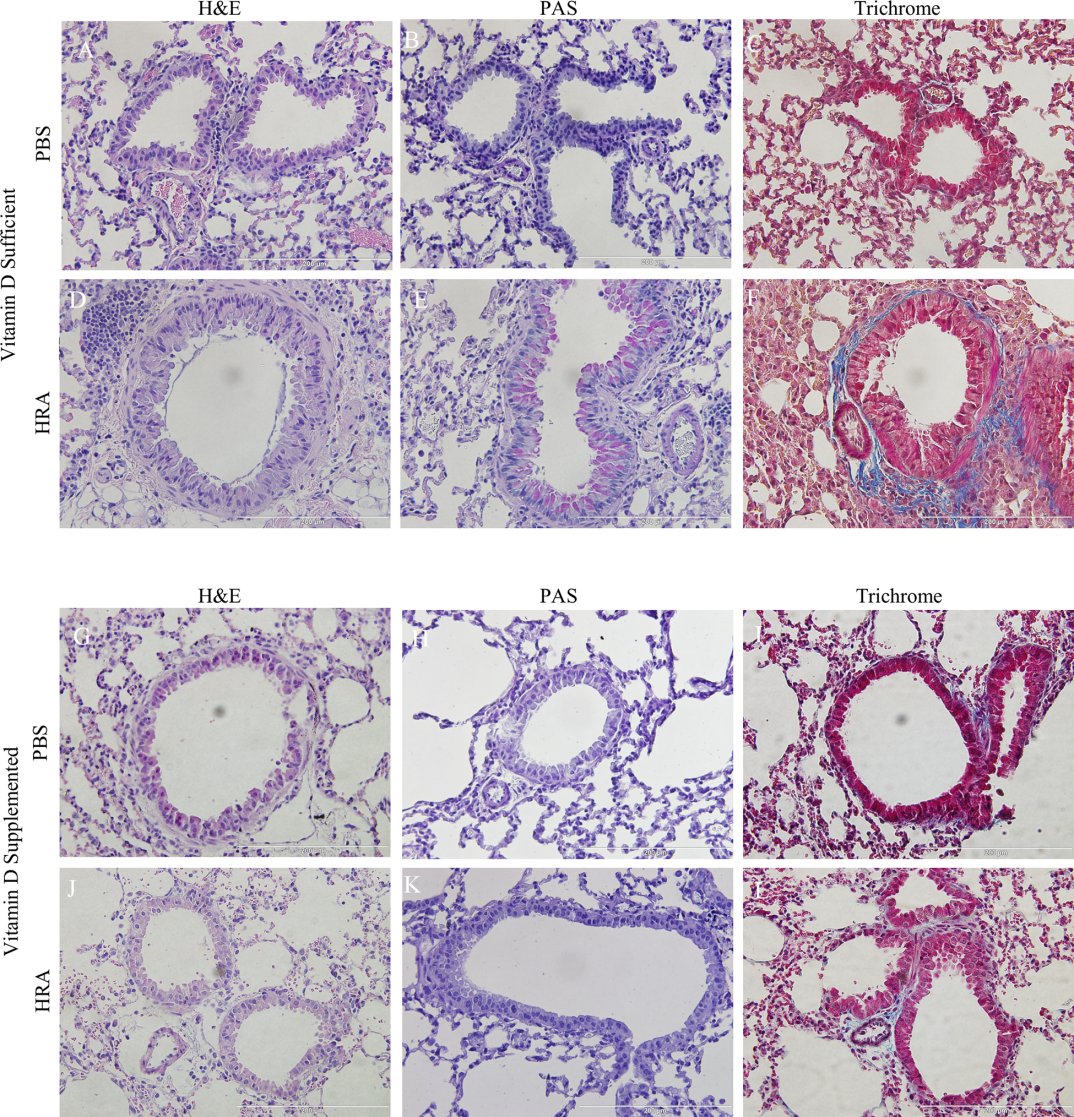
Effect of vitamin D on lung histology in PBS and HRA-sensitized mice. Histology of the thin sections of the lungs of vitamin D-sufficient (A-F) and vitamin D-supplemented (G-L) mice from PBS and HRA groups is shown. H&E staining (A, D, G, J) shows morphological changes with eosin staining the cytoplasm pink and the blue nucleus stained with hematoxylin; PAS staining (B, E. H, K) shows mucus staining in pink; Trichrome staining (C, F, I, L) indicates collagen deposition stained in blue. The histological pictures are the representatives of seven independent animals (n = 7) in each experimental group.

**Fig 5:**
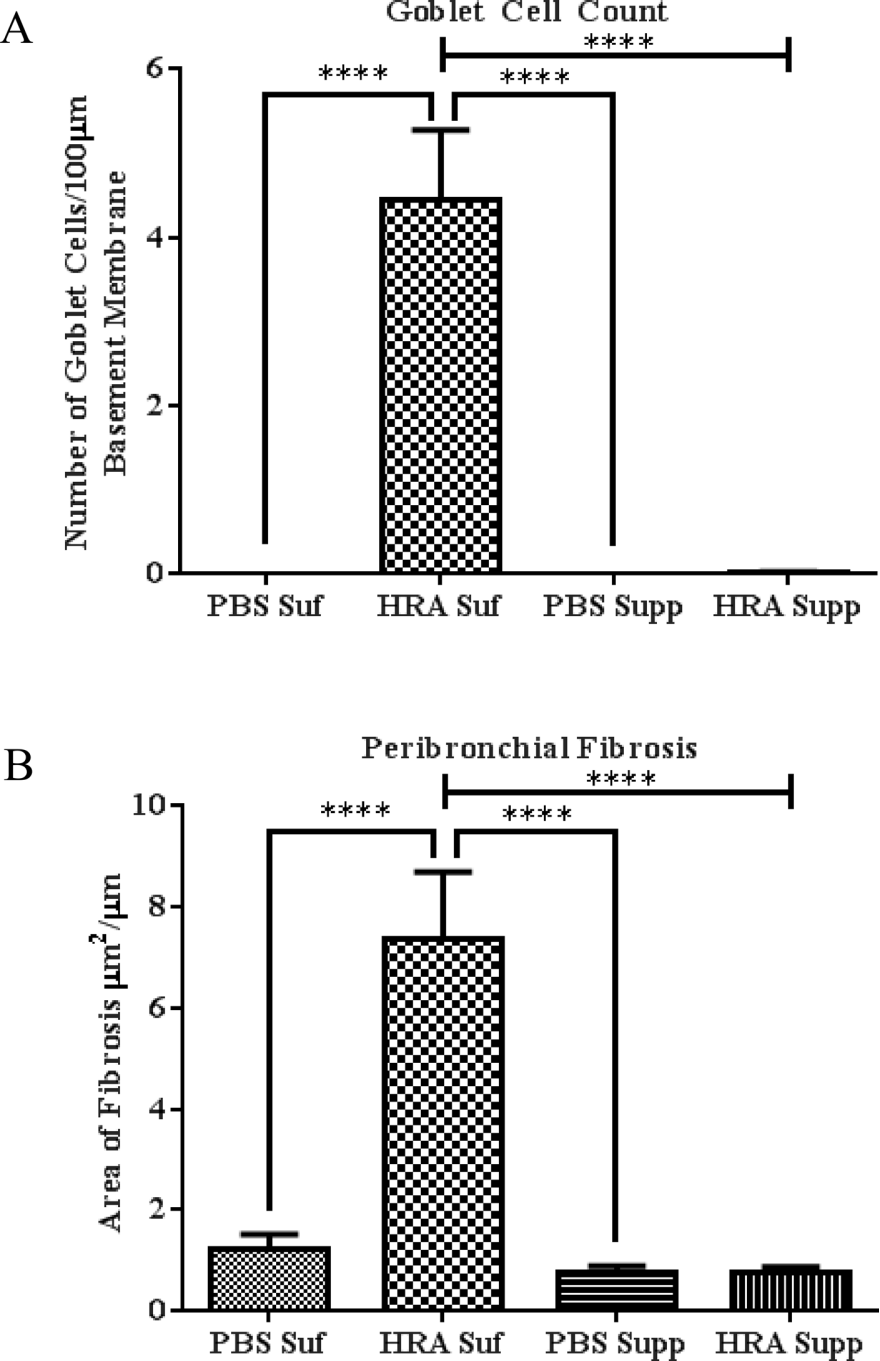
Morphometric analysis of lung tissues of PBS and HRA-sensitized and challenged mice. (A) Goblet cells were quantified by counting the number of PAS-positive goblet cells per 100 μm of bronchial basal lamina as determined by tracing the airway in the ImageJ analysis software. (B) Trichrome staining was used to evaluate subepithelial fibrosis in the peribronchial area. The trichrome-stained fibrotic area was outlined and quantified using ImageJ and expressed as area of fibrosis (μm^2^) per micrometer of basal lamina. The results are presented as mean ± SEM of 7 mice per group with 10 measurements per mouse, ****p <0.0001.

After tracheostomy animals were sacrificed; lungs were harvested and sectioned, followed by staining with H&E, PAS, and trichrome stains to examine histological hallmarks of asthmatic airways. Airways of vitamin D-sufficient PBS mice displayed normal parenchyma (Fig 4A), with no mucus staining (Fig 4B), and little collagen staining around the respiratory epithelium (Fig 4C). Vitamin D-sufficient HRA mice exhibited signs of mild airway remodeling, showing airway epithelial cell hypertrophy (Fig 4D), mild mucus staining (Fig 4E), and collagen deposition (Fig 4F). However, in vitamin D-supplemented mice, features of airway remodeling were absent in both PBS and HRA groups (Fig 4G and 4J). Also, there was no positive staining to mucus (Fig 4H and 4K) and collagen (Fig 4I and 4L), as determined by periodic acid Schiff (PAS) (Fig 5A) and trichrome stains (Fig 5B), respectively.

### Effect of Vitamin D on the Expression of E-cadherin, Vimentin, and N-cadherin in the Lungs of HRA-Sensitized and Challenged Mice

**Fig 6:**
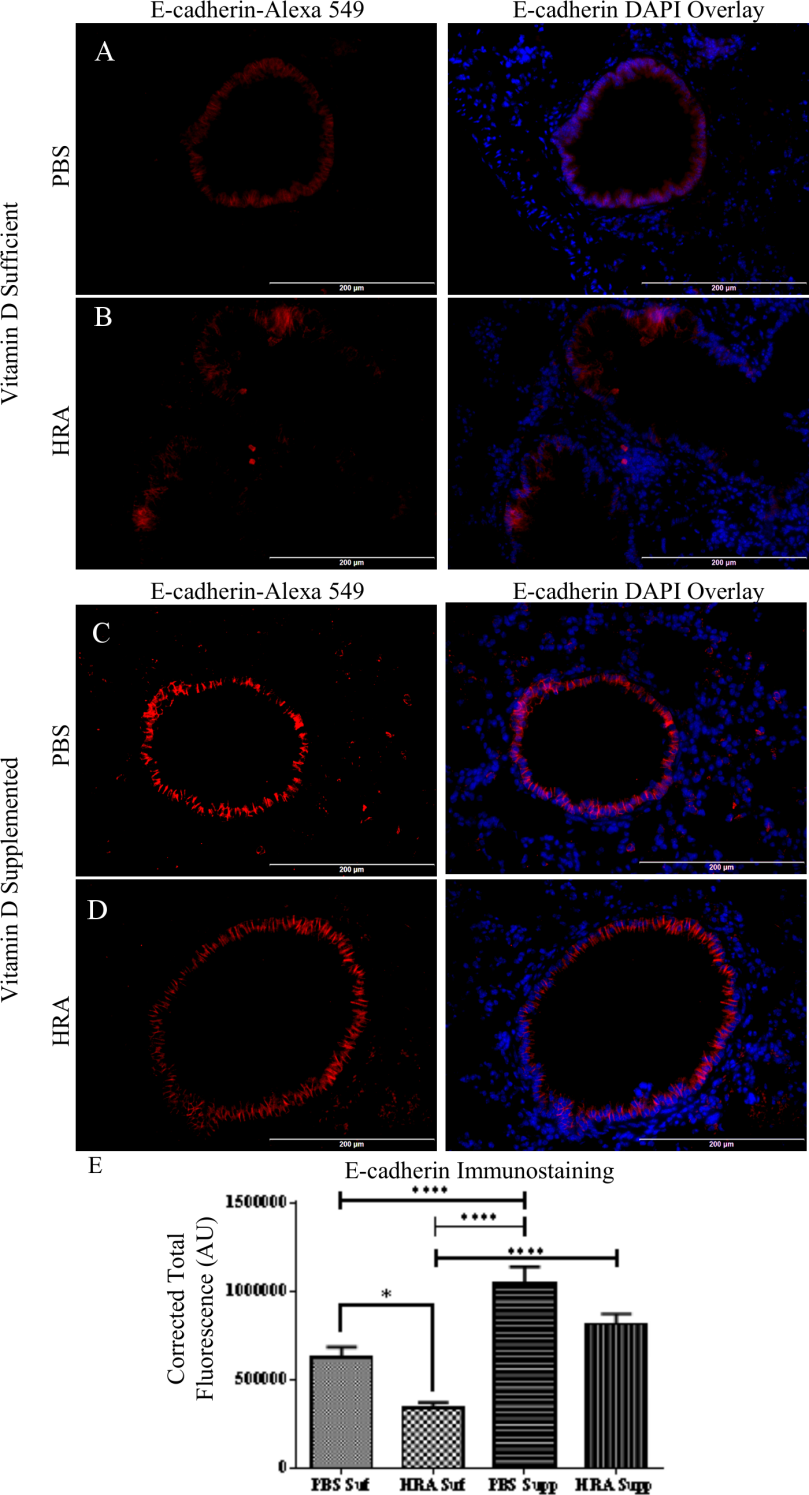
Effect of vitamin D on E-cadherin expression in lung epithelium of PBS and HRA-sensitized and challenged mice. (A, B) Immunofluorescence image shows immunostaining of E-cadherin in the lungs of vitamin D-sufficient PBS control mice compared to vitamin D-sufficient HRA-sensitized and challenged mice (40× magnification). Right panels: Sections stained with rabbit anti-E-cadherin antibody and goat anti-rabbit Alexa Fluor 549 as secondary antibody; Left panels: Merged Alexa Fluor 549 and DAPI used to stain the nuclei; (C, D) Expression of E-cadherin in the lungs of vitamin D-supplemented PBS control mice compared to vitamin D-supplemented HRA-sensitized and challenged mice (40x magnification); (E) Corrected total fluorescence (CTF) of proteins in the airways was measured in arbitrary units (AU) using ImageJ software. The results are presented as mean ± SEM of 7 mice per group with 10 measurements per mouse, *p <0.05, ****p <0.0001.

Expression of E-cadherin was observed in the lung tissue of PBS control mice in both vitamin D-sufficient and supplemented groups (Fig 6A and 6C). The epithelium in the HRA-sensitized and challenged mice demonstrated significant decreased expression of E-cadherin (Fig 6B) as determined using ImageJ software. E-cadherin expression was protected by the vitamin D-supplemented diet, as observed in the vitamin D-supplemented HRA-sensitized and challenged mice (Figs 6D and 5E).

**Fig 7:**
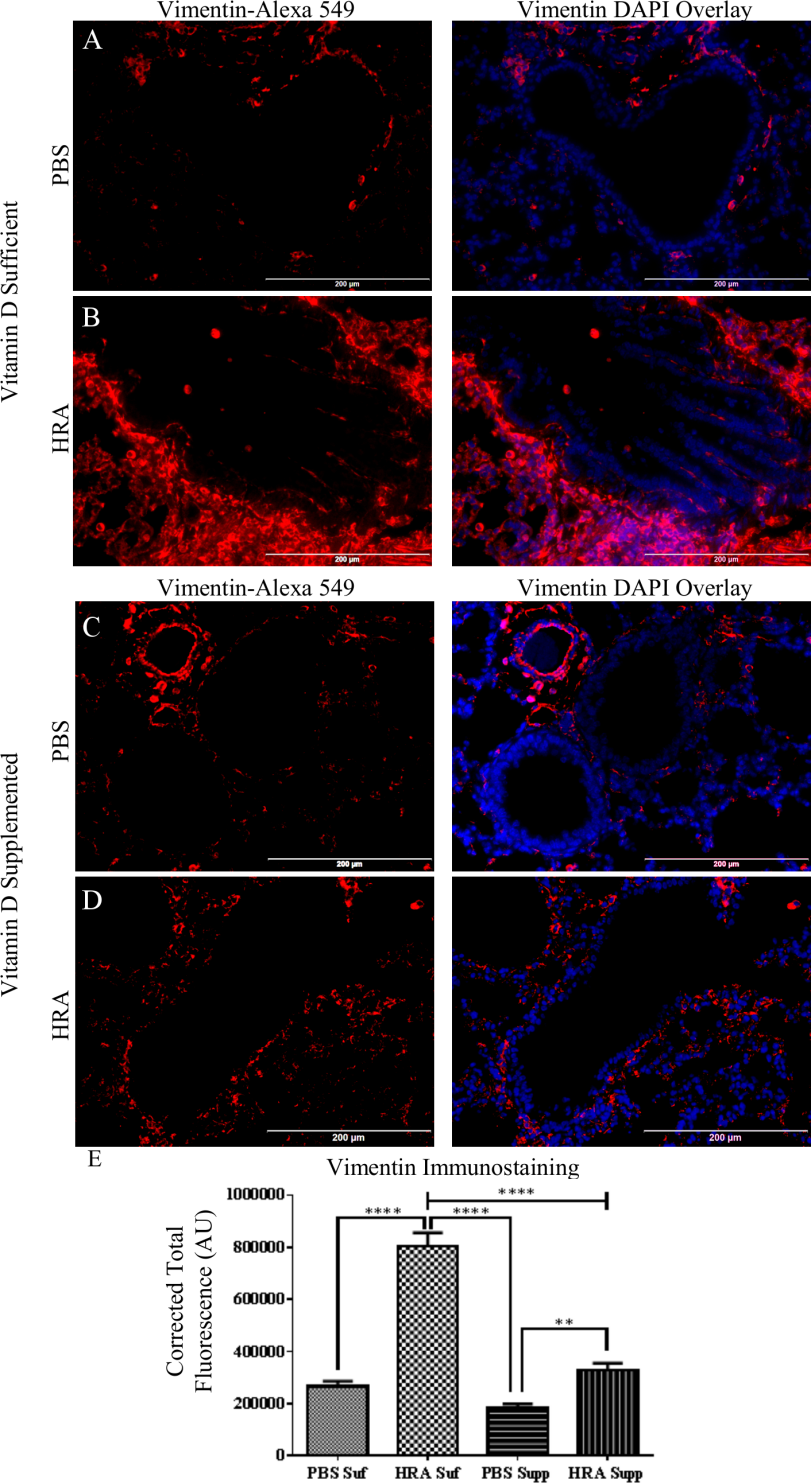
Effect of vitamin D on vimentin expression in lung epithelium of PBS and HRA-sensitized and challenged mice. (A, B) Immunofluorescence image shows the immunostaining of vimentin, in the lungs of vitamin D-sufficient PBS control mice compared to vitamin D-sufficient HRA-sensitized and challenged mice (40× magnification). Right panels: Sections stained using rabbit anti-vimentin antibody and goat anti-rabbit Alexa Fluor 549 as secondary antibody; Left panels: Merged Alexa Fluor 549 and DAPI used to stain the nuclei. (C, D) Expression of vimentin in the lungs of vitamin D-supplemented PBS control mice compared to vitamin D-supplemented HRA-sensitized and challenged mice (40x magnification). (E) CTF of proteins in the airways was measured in AU using ImageJ software. The results are presented as mean ± SEM of 7 mice per group with 10 measurements per mouse, **p <0.01, ****p <0.0001.

The expression of vimentin in the lung tissue was marginally expressed in the peribronchial area in the vitamin D-sufficient PBS mice (Fig 7A and 7C). However, in the vitamin D-sufficient HRA-sensitized and challenged mice, vimentin expression was prominently increased in expression and found to be within the epithelial layer of the airway (Fig 7B). Vimentin expression was found to be significantly increased in vitamin D-supplemented HRA-sensitized and challenged mice (Fig 7D) compared to vitamin D-supplemented PBS-sensitized and challenged mice (Fig 7C). However, the expression of vimentin in the airway epithelium of vitamin D-sufficient HRA mice was significantly greater than in the vitamin D-supplemented HRA-sensitized and challenged mice (Fig 7E).

**Fig 8:**
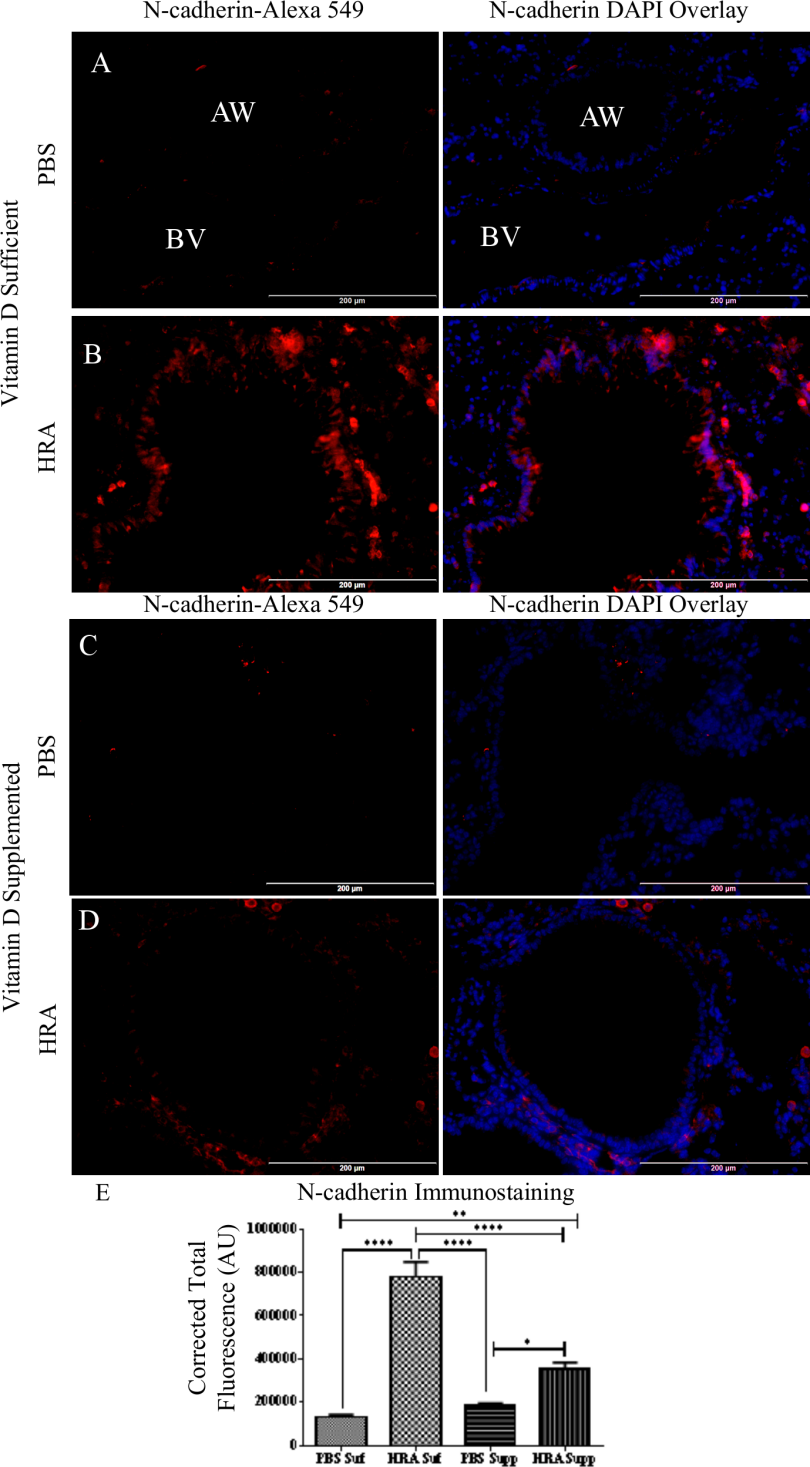
Effect of vitamin D on N-cadherin expression in lung epithelium of PBS and HRA-sensitized mice. (A, B) Immunofluorescence image shows immunostaining of N-cadherin, in the lungs of vitamin D-sufficient PBS control mice compared to vitamin D- sufficient HRA-sensitized and challenged mice (40× magnification); AW: Airway BV: Blood vessel. Right panels: Sections stained using rabbit anti-N-cadherin antibody and goat anti-rabbit Alexa Fluor 549 as secondary antibody; Left panels: Merged Alexa Fluor 549 and DAPI used to stain the nuclei; (C, D) Expression of N-cadherin in the lungs of vitamin D-supplemented PBS control mice compared to vitamin D-supplemented HRA-sensitized and challenged mice (40x magnification); (E) Corrected total fluorescence (CTF) of proteins in the airways was measured in AU using ImageJ software. The results are presented as mean ± SEM of 7 mice per group with 10 measurements per mouse, *p <0.05, ** p <0.01, ****p <0.0001.

N-cadherin expression was minimal in the lamina propria in both groups of PBS mice (Fig 8A and 8C). N-cadherin was expressed in the blood vessels of the airway (Fig 8A), consistent with the reports of N-cadherin being expressed normally in vascular smooth muscle cells [18]. N-cadherin was expressed in vitamin D-sufficient HRA-sensitized and challenged mice (Fig 8B). This expression was decreased by vitamin D supplementation, as determined by vitamin D-supplemented HRA-sensitized and challenged mice (Fig 8D). However, vitamin D supplementation was not enough to prevent N-cadherin expression by HRA, as evidenced by comparing with the vitamin D- supplemented PBS-sensitized and challenged mice (Fig 8E).

These results confirm our earlier studies in bronchial epithelial cells that in an inflammatory environment, vitamin D increases the expression of E-cadherin, and decreases the expression of vimentin and N-cadherin [19].

## Discussion

Structural changes in the airway epithelium lead to abnormal lung function and AHR. Physical alterations in the asthmatic epithelium include loss of E-cadherin [20] and induction of myofibroblast markers, N-cadherin and vimentin [21]. Myofibroblasts are a part of the wound healing response [22] and have been implicated in the invasion of cancer cells into the epithelium compartment of the colon [23]. Damage to the airway epithelium from allergens or other environmental factors can lead to the release of inflammatory mediators that promote migration of the epithelial cell to allow for wound repair. Myofibroblasts can be derived from epithelial cells through induction of EMT. EMT in wound healing is a normal biological procedure. However, in the context of an allergic response, this process becomes detrimental to the airway allowing transient properties to become permanent.

Corticosteroid treatment has been found to be ineffective against TGF-β mediated differentiation of myofibroblasts [24], [25]. For that reason alternates to anti-inflammatory treatments are required. Vitamin D has emerged as an effective anti-inflammatory agent and has been found to be effective in asthma, specifically in glucocorticoid-resistant asthma [26]. However, we still do not fully understand the underlying cellular and molecular mechanisms of vitamin D effects in the pathogenesis and/or treatment of asthma. *In vitro* studies have observed anti-inflammatory effects of vitamin D in airway smooth muscle cells [27], fibroblasts [28], and epithelial cells [29]. *In vivo* studies also suggest improvement in lung function and improved structural integrity in the lung with vitamin D administration [30].

In this study, we found that vitamin D decreased the amount of IgE in both serum and in the BALF. This is consistent with the reports of high concentrations of serum total IgE in asthmatic patients with vitamin D deficiency [31]. While vitamin D was found to have no effect on total IgE in a vitamin D-deficient OVA-mouse model [32], supplementation of vitamin D in the diet of vitamin D-deficient mice has been reported to decrease eosinophil and neutrophil numbers in the BALF [33]. In other studies, administration of 1,25(OH)_2_D_3_ suppressed allergen-specific IgE levels, eosinophilic airway inflammation, AHR, along with reduced IL-5 and IL-13 levels in BALF and reduced production of Th2 cytokines by T-cells [34]. Overall, most of the findings, including ours in this study, support the beneficial effect of vitamin D in attenuating allergic airway inflammation and asthma.

The immunomodulatory properties of vitamin D have been associated with several inflammatory diseases [35]. In asthma, vitamin D deficiency has been linked with worse outcomes [36–38]. The use of vitamin D supplementation as a form of therapy in patients with asthma has not been without controversy. A randomized double-blind study analyzing children on glucocorticoid steroid with or without 500 IU/day of vitamin D_3_ found significant improvement in the absolute and predicted forced expiratory volume in 1 second (FEV_1_) as well as decreased exacerbations [39]. Another study in asthmatic subjects using 0.25 μg calcitriol capsules twice daily demonstrated significant improvement in the absolute and percent predicted in FEV_1_ [40]. Vitamin D supplementation was also found to improve FEV_1_ in 24 weeks in patients on glucocorticoid steroids after receiving an initial bolus of 100,000 IU followed by 50,000 IU weekly [26]. Conversely, another recent study also evaluating patients on glucocorticoid steroids with or without an initial dose of 100,000 IU of vitamin D_3_ followed by a daily dose of 4,000 IU found no difference in asthma control or quality of life. Results of this trial also indicated that the total inhaled corticosteroid dose was lower in the vitamin D_3_ treatment group [41]. Vitamin D insufficiency in patients with chronic kidney disease can be combated by a weekly dose of 10,000 IU oral cholecalciferol supplement, raising 25-hydroxyvitamin D levels twofold [42]. The guidelines of the Institute of Medicine classify risk of vitamin D deficiency when serum levels of 25(OH)D are below 20 ng/ml (50 nM) [43]. Many clinical laboratories continue reporting a value of 20 ng/ml as inadequate and that 21–29 ng/ml (52.5–72.5 nM) as insufficient levels [44]. However, in patients at risk for vitamin D deficiency, the Institute of Medicine recommended a daily intake of 800 IU of vitamin D3 to raise the blood level of 25(OH)D. The Endocrine Society Task Force has proposed that 1,500–2,000 IU of vitamin D daily is required to raise blood 25(OH)D levels to a sufficient range [45]. In this study, we administered 10,000 IU vitamin D3 to mice to achieve the serum levels of 25(OH)D that is generally found in human with 3,000–4,000 IU vitamin D3 supplementation without any toxicity. The difference in the magnitude of increased serum 25(OH)D levels in mice and human could be related to differential rate of vitamin D metabolism. Nonetheless, the findings support the role of supplemental vitamin D in the attenuation of hallmark features of allergic asthma.

We report findings that vitamin D supplementation is associated with lower AHR to methacholine with decreased intensity of inflammation, decreased bronchoconstrictory response to methacholine, and decreased serum and BAL fluid IgE levels compared to vitamin D-sufficiency in HRA-sensitized and challenged mice. Increased inflammation in the lung was also displayed in the cells recovered from the BAL fluid with increased macrophages, lymphocytes, and eosinophils in the vitamin D-sufficient compared to vitamin D supplemented HRA-sensitized and challenged mice. These results are consistent with previously published results where vitamin D supplementation, while not fully reversing the effects of allergic airway inflammation, did reduce AHR, airway remodeling and BALF cytokines levels [30].

## Conclusions

The findings in this study suggest that vitamin D supplementation reduces the effect of allergic airway inflammation in a mouse model with nasal aerosolization of clinically relevant allergens. This study further indicates a role for the epithelium in mediating airway remodeling in asthma. However, further research will need to be conducted in order to elucidate the effect of vitamin D on antigen presentation by dendritic cells to T cells and the immune response in this mouse model. These results also suggest that the epithelium is a therapeutic target for the prevention of airway remodeling in asthma.

## References

[1] Lemanske RF, Busse WW. Asthma. J Allergy Clin Immunol. 2003 2;111(2 Suppl):S502–19. 12592297 10.1067/mai.2003.94 PMC7112293

[2] Kelly MM, O’Connor TM, Leigh R, Otis J, Gwozd C, Gauvreau GM, et al Effects of budesonide and formoterol on allergen-induced airway responses, inflammation, and airway remodeling in asthma. J Allergy Clin Immunol. 2010 2;125(2):349–56.e13. 10.1016/j.jaci.2009.09.011 19969339

[3] Berair R, Brightling CE. Asthma Therapy and Its Effect on Airway Remodelling. Drugs. 2014 7 24; 10.1007/s40265-014-0250-4 25056652

[4] Bergeron C, Tulic MK, Hamid Q. Airway remodelling in asthma: from benchside to clinical practice. Can Respir J J Can Thorac Soc. 2010 8;17(4):e85–93. 10.1155/2010/318029 PMC2933777 20808979

[5] Lambrecht BN, Hammad H. The airway epithelium in asthma. Nat Med. 2012 5;18(5):684–92. 10.1038/nm.2737 22561832

[6] Bergeron C, Al-Ramli W, Hamid Q. Remodeling in asthma. Proc Am Thorac Soc. 2009 5 1;6(3):301–5. 10.1513/pats.200808-089RM 19387034

[7] Berraies A, Hamzaoui K, Hamzaoui A. Link between vitamin D and airway remodeling. J Asthma Allergy. 2014 4 1;7:23–30. 10.2147/JAA.S46944 24729717 PMC3979801

[8] Yawn J, Lawrence LA, Carroll WW, Mulligan JK. Vitamin D for the treatment of respiratory diseases: is it the end or just the beginning? J Steroid Biochem Mol Biol. 2015 4;148:326–37. 10.1016/j.jsbmb.2015.01.017 25625665

[9] Nials AT, Uddin S. Mouse models of allergic asthma: acute and chronic allergen challenge. Dis Model Mech. 2008;1(4–5):213–20. 10.1242/dmm.000323 19093027 PMC2590830

[10] Johnson JR, Roos A, Berg T, Nord M, Fuxe J. Chronic Respiratory Aeroallergen Exposure in Mice Induces Epithelial-Mesenchymal Transition in the Large Airways. Samakovlis C, editor. PLoS ONE. 2011 1 20;6(1):e16175 10.1371/journal.pone.0016175 21283768 PMC3024415

[11] Fan M, Jamal Mustafa S. Role of adenosine in airway inflammation in an allergic mouse model of asthma. Int Immunopharmacol. 2006 1;6(1):36–45. 16332511 10.1016/j.intimp.2005.07.008

[12] Sorkness RL, Herricks KM, Szakaly RJ, Lemanske RF, Rosenthal LA. Altered allergen-induced eosinophil trafficking and physiological dysfunction in airways with preexisting virus-induced injury. Am J Physiol Lung Cell Mol Physiol. 2007 1;292(1):L85–91. 16905639 10.1152/ajplung.00234.2006

[13] Shao Z, Bharadwaj AS, McGee HS, Makinde TL, Agrawal DK. Flt-3 Ligand Increases a Lung Dendritic Cell Subset with Regulatory Properties in allergic airway inflammation. J Allergy Clin Immunol. 2009 4;123(4):917–24.e2. 10.1016/j.jaci.2009.01.052 19348927 PMC2690643

[14] Locke NR, Royce SG, Wainewright JS, Samuel CS, Tang ML. Comparison of Airway Remodeling in Acute, Subacute, and Chronic Models of Allergic Airways Disease. Am J Respir Cell Mol Biol. 2007 5 1;36(5):625–32. 17237192 10.1165/rcmb.2006-0083OC

[15] Ge XN, Bahaie NS, Kang BN, Hosseinkhani MR, Ha SG, Frenzel EM, et al Allergen-Induced Airway Remodeling Is Impaired in Galectin-3–Deficient Mice. J Immunol. 2010 7 15;185(2):1205–14. 10.4049/jimmunol.1000039 20543100 PMC2918241

[16] Jensen EC. Quantitative Analysis of Histological Staining and Fluorescence Using ImageJ. Anat Rec. 2013 3 1;296(3):378–81. 10.1002/ar.22641 23382140

[17] Kim M-H, Jee J-H, Park S, Lee M-S, Kim K-W, Lee M-K. Metformin enhances glucagon-like peptide 1 via cooperation between insulin and Wnt signaling. J Endocrinol. 2014 2;220(2):117–28. 10.1530/JOE-13-0381 24233023

[18] Sun Z, Parrish AR, Hill MA, Meininger GA. N-cadherin, A Vascular Smooth Muscle Cell–Cell Adhesion Molecule: Function and Signaling for Vasomotor Control. Microcirculation. 2014 4 1;21(3):208–18. 10.1111/micc.12123 24521477

[19] Fischer KD, Agrawal DK. Vitamin D regulating TGF-β induced epithelial-mesenchymal transition. Respir Res. 2014;15(1):146. 25413472 10.1186/s12931-014-0146-6 PMC4245846

[20] Hackett T-L, de Bruin HG, Shaheen F, van den Berge M, van Oosterhout AJ, Postma DS, et al Caveolin-1 controls airway epithelial barrier function. Implications for asthma. Am J Respir Cell Mol Biol. 2013 10;49(4):662–71. 10.1165/rcmb.2013-0124OC 23742006

[21] Johnson JR, Nishioka M, Chakir J, Risse P-A, Almaghlouth I, Bazarbashi AN, et al IL-22 contributes to TGF-β1-mediated epithelial-mesenchymal transition in asthmatic bronchial epithelial cells. Respir Res. 2013;14:118 10.1186/1465-9921-14-118 24283210 PMC4176096

[22] Micallef L, Vedrenne N, Billet F, Coulomb B, Darby IA, Desmoulière A. The myofibroblast, multiple origins for major roles in normal and pathological tissue repair. Fibrogenesis Tissue Repair. 2012;5(Suppl 1 Proceedings of Fibroproliferative disorders: from biochemical analysis to targeted therapiesPetro E Petrides and David Brenner):S5 10.1186/1755-1536-5-S1-S5 23259712 PMC3368789

[23] De Wever O, Westbroek W, Verloes A, Bloemen N, Bracke M, Gespach C, et al Critical role of N-cadherin in myofibroblast invasion and migration in vitro stimulated by colon-cancer-cell-derived TGF-β or wounding. J Cell Sci. 2004 9 15;117(20):4691–703. 15331629 10.1242/jcs.01322

[24] Zhang M, Zhang Z, Pan H-Y, Wang D-X, Deng Z-T, Ye X-L. TGF-beta1 induces human bronchial epithelial cell-to-mesenchymal transition in vitro. Lung. 2009 6;187(3):187–94. 10.1007/s00408-009-9139-5 19252942

[25] Doerner AM, Zuraw BL. TGF-β1 induced epithelial to mesenchymal transition (EMT) in human bronchial epithelial cells is enhanced by IL-1β but not abrogated by corticosteroids. Respir Res. 2009;10(1):100. 19857272 10.1186/1465-9921-10-100 PMC2774671

[26] Arshi S, Fallahpour M, Nabavi M, Bemanian MH, Javad-Mousavi SA, Nojomi M, et al The effects of vitamin D supplementation on airway functions in mild to moderate persistent asthma. Ann Allergy Asthma Immunol. 2014 10;113(4):404–9. 10.1016/j.anai.2014.07.005 25091714

[27] Agrawal T, Gupta GK, Agrawal DK. Calcitriol decreases expression of importin α3 and attenuates RelA translocation in human bronchial smooth muscle cells. J Clin Immunol. 2012 10;32(5):1093–103. 10.1007/s10875-012-9696-x 22526597 PMC3444658

[28] Ramirez AM, Wongtrakool C, Welch T, Steinmeyer A, Zügel U, Roman J. Vitamin D inhibition of pro-fibrotic effects of transforming growth factor β1 in lung fibroblasts and epithelial cells. J Steroid Biochem Mol Biol. 2010 2;118(3):142–50. 10.1016/j.jsbmb.2009.11.004 19931390 PMC2821704

[29] Hansdottir S, Monick MM, Lovan N, Powers L, Gerke A, Hunninghake GW. Vitamin D decreases respiratory syncytial virus induction of NF-kappaB-linked chemokines and cytokines in airway epithelium while maintaining the antiviral state. J Immunol Baltim Md 1950. 2010 1 15;184(2):965–74. 10.4049/jimmunol.0902840 PMC3035054 20008294

[30] Agrawal T, Gupta GK, Agrawal DK. Vitamin D supplementation reduces airway hyperresponsiveness and allergic airway inflammation in a murine model. Clin Exp Allergy J Br Soc Allergy Clin Immunol. 2013 6;43(6):672–83. 10.1111/cea.12102 PMC3671499 23711130

[31] Hatami G, Ghasemi K, Motamed N, Firoozbakht S, Movahed A, Farrokhi S. Relationship between Vitamin D and Childhood Asthma: A Case–Control Study. Iran J Pediatr. 2014 12;24(6):710–4. 26019776 PMC4442832

[32] Gorman S, Tan DHW, Lambert MJM, Scott NM, Judge MA, Hart PH. Vitamin D3 deficiency enhances allergen-induced lymphocyte responses in a mouse model of allergic airway disease. Pediatr Allergy Immunol. 2012 2 1;23(1):83–7. 10.1111/j.1399-3038.2011.01146.x 22283404

[33] Gorman S, Weeden CE, Tan DHW, Scott NM, Hart J, Foong RE, et al Reversible Control by Vitamin D of Granulocytes and Bacteria in the Lungs of Mice: An Ovalbumin-Induced Model of Allergic Airway Disease. PLoS ONE [Internet]. 2013 6 24 [cited 2015 Jun 5];8(6). Available from: http://www.ncbi.nlm.nih.gov/pmc/articles/PMC3691156/10.1371/journal.pone.0067823 PMC3691156 23826346

[34] Taher YA, Esch BCAM van, Hofman GA, Henricks PAJ, Oosterhout AJM van. 1α,25-Dihydroxyvitamin D3 Potentiates the Beneficial Effects of Allergen Immunotherapy in a Mouse Model of Allergic Asthma: Role for IL-10 and TGF-β. J Immunol. 2008 4 15;180(8):5211–21. 18390702 10.4049/jimmunol.180.8.5211

[35] Yin K, Agrawal DK. Vitamin D and inflammatory diseases. J Inflamm Res. 2014;7:69–87. 10.2147/JIR.S63898 24971027 PMC4070857

[36] Bener A, Ehlayel MS, Tulic MK, Hamid Q. Vitamin D Deficiency as a Strong Predictor of Asthma in Children. Int Arch Allergy Immunol. 2012;157(2):168–75. 10.1159/000323941 21986034

[37] Foong RE, Shaw NC, Berry LJ, Hart PH, Gorman S, Zosky GR. Vitamin D deficiency causes airway hyperresponsiveness, increases airway smooth muscle mass, and reduces TGF-β expression in the lungs of female BALB/c mice. Physiol Rep. 2014;2(3):e00276 10.1002/phy2.276 24760528 PMC4002254

[38] Freishtat RJ, Iqbal SF, Pillai DK, Klein CJ, Ryan LM, Benton AS, et al High Prevalence of Vitamin D Deficiency among Inner-City African American Youth with Asthma in Washington, DC. J Pediatr. 2010 6;156(6):948–52. 10.1016/j.jpeds.2009.12.033 20236657 PMC3328513

[39] Majak P, Olszowiec-Chlebna M, Smejda K, Stelmach I. Vitamin D supplementation in children may prevent asthma exacerbation triggered by acute respiratory infection. J Allergy Clin Immunol. 2011 5;127(5):1294–6. 10.1016/j.jaci.2010.12.016 21315433

[40] Nanzer AM, Chambers ES, Ryanna K, Freeman AT, Colligan G, Richards DF, et al The effects of calcitriol treatment in glucocorticoid-resistant asthma. J Allergy Clin Immunol. 2014 6;133(6):1755–7.e4. 10.1016/j.jaci.2014.03.015 24786237

[41] Castro M, King TS, Kunselman SJ, et al Effect of vitamin d3 on asthma treatment failures in adults with symptomatic asthma and lower vitamin d levels: The vida randomized clinical trial. JAMA. 2014 5 28;311(20):2083–91. 10.1001/jama.2014.5052 24838406 PMC4217655

[42] Cupisti A, Vigo V, Baronti ME, D’Alessandro C, Ghiadoni L, Egidi MF. Vitamin D status and cholecalciferol supplementation in chronic kidney disease patients: an Italian cohort report. Int J Nephrol Renov Dis. 2015 11 19;8:151–7. 10.2147/IJNRD.S90968 PMC4657801 26640388

[43] Rosen CJ, Abrams SA, Aloia JF, Brannon PM, Clinton SK, Durazo-Arvizu RA, et al IOM committee members respond to Endocrine Society vitamin D guideline. J Clin Endocrinol Metab. 2012 4;97(4):1146–52. 10.1210/jc.2011-2218 22442278 PMC5393439

[44] Muehleisen B, Gallo RL. Vitamin D in allergic disease: shedding light on a complex problem. J Allergy Clin Immunol. 2013 2;131(2):324–9. 10.1016/j.jaci.2012.12.1562 23374263

[45] Holick MF, Binkley NC, Bischoff-Ferrari HA, Gordon CM, Hanley DA, Heaney RP, et al Evaluation, treatment, and prevention of vitamin D deficiency: an Endocrine Society clinical practice guideline. J Clin Endocrinol Metab. 2011 7;96(7):1911–30. 10.1210/jc.2011-0385 21646368

